# *N*,*N*′-(2-Hy­droxy­propane-1,3-di­yl)bis­(2-hy­droxy­benzamide) monohydrate

**DOI:** 10.1107/S1600536813026184

**Published:** 2013-09-28

**Authors:** Sihem Yebedri, Samira Louhibi, Sofiane Bouacida, Ali Ourari, Thierry Roisnel

**Affiliations:** a Laboratoire de Chimie Inorganiue et d’Environment, Université def Tlemcen, BP 119, Tlemcen, 13 000, Algeria; b Unité de Recherche de Chimie de l’Environnement et Moléculaire Structurale, CHEMS, Université Mentouri-Constantine, 25000 , Algeria; c Laboratoire d’Electrochimie, d’Ingénierie Moléculaire et de Catalyse Redox (LEIMCR), Faculté des Sciences de l’Ingénieur, Université Farhat Abbas, Sétif, 19000 , Algeria; d Centre de Difractométrie X, UMR 6226 CNRS Unité Sciences Chimiques de Rennes, Université de Rennes I, 263 Avenue du Général Leclerc, 35042 Rennes, France

## Abstract

In the title hydrate, C_17_H_18_N_2_O_5_·H_2_O, the complete organic mol­ecule is generated by a crystallographic mirror plane with one C and one O atom lying on the mirror plane. The O atom of the water mol­ecule has *m* site symmetry. Two symmetry-related intra­molecular O—H⋯O hydrogen bonds complete *S*(6) rings in the organic mol­ecule. In the crystal, the components are linked into (010) sheets by O—H⋯O and N—H⋯O hydrogen bonds.

## Related literature

For the synthesis of similar compounds and their complexes see: Kumar & Debashis (2006 ▶); Azam *et al.* (2012 ▶); Sarkar (1999 ▶); Louhibi *et al.* (2007 ▶); Kui *et al.* (2009 ▶).
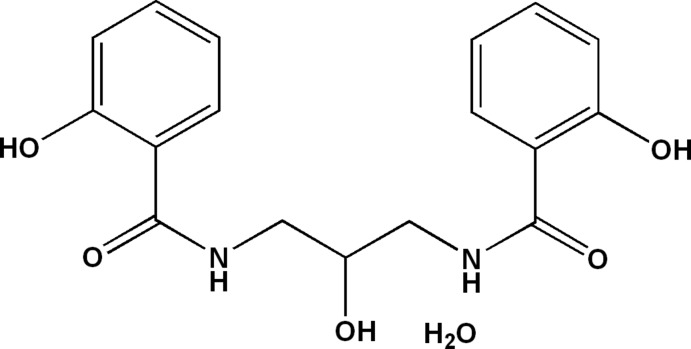


## Experimental

### 

#### Crystal data

C_17_H_18_N_2_O_5_·H_2_O
*M* *_r_* = 348.35
Orthorhombic, 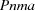

*a* = 12.8969 (10) Å
*b* = 28.001 (2) Å
*c* = 4.5330 (4) Å
*V* = 1637.0 (2) Å^3^
*Z* = 4
Mo *K*α radiation
μ = 0.11 mm^−1^
*T* = 150 K
0.38 × 0.12 × 0.04 mm

#### Data collection

Bruker APEXII diffractometer
Absorption correction: multi-scan (*SADABS*; Bruker, 2011 ▶) *T*_min_ = 0.877, *T*_max_ = 0.996
7880 measured reflections
1904 independent reflections
1195 reflections with *I* > 2σ(*I*)
*R*_int_ = 0.062

#### Refinement

*R*[*F* ^2^ > 2σ(*F* ^2^)] = 0.048
*wR*(*F* ^2^) = 0.123
*S* = 1.04
1904 reflections
124 parameters
H atoms treated by a mixture of independent and constrained refinement
Δρ_max_ = 0.21 e Å^−3^
Δρ_min_ = −0.23 e Å^−3^

### 

Data collection: *APEX2* (Bruker, 2011 ▶); cell refinement: *SAINT* (Bruker, 2011 ▶); data reduction: *SAINT*; program(s) used to solve structure: *SIR2002* (Burla *et al.*, 2005 ▶); program(s) used to refine structure: *SHELXL97* (Sheldrick, 2008 ▶); molecular graphics: *ORTEP-3 for Windows* (Farrugia, 2012 ▶) and *DIAMOND* (Brandenburg & Berndt, 2001 ▶); software used to prepare material for publication: *WinGX* (Farrugia, 2012 ▶) and *CRYSCAL* (T. Roisnel, local program).

## Supplementary Material

Crystal structure: contains datablock(s) I. DOI: 10.1107/S1600536813026184/hb7142sup1.cif

Structure factors: contains datablock(s) I. DOI: 10.1107/S1600536813026184/hb7142Isup2.hkl

Supplementary material file. DOI: 10.1107/S1600536813026184/hb7142Isup3.cml

Additional supplementary materials:  crystallographic information; 3D view; checkCIF report

## Figures and Tables

**Table 1:** Hydrogen-bond geometry (Å, °)

*D*—H⋯*A*	*D*—H	H⋯*A*	*D*⋯*A*	*D*—H⋯*A*
O1—H1⋯O1*W* ^i^	0.96 (3)	1.82 (3)	2.778 (3)	179 (3)
O1*W*—H1*W*⋯O6^ii^	0.92 (2)	1.92 (2)	2.827 (2)	174 (2)
O13—H13⋯O6	0.84	1.75	2.500 (2)	147
N4—H4⋯O13^iii^	0.88	2.07	2.915 (2)	160

Symmetry codes: (i) 
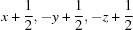
; (ii) 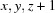; (iii) 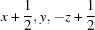.

**Fig. 1.**
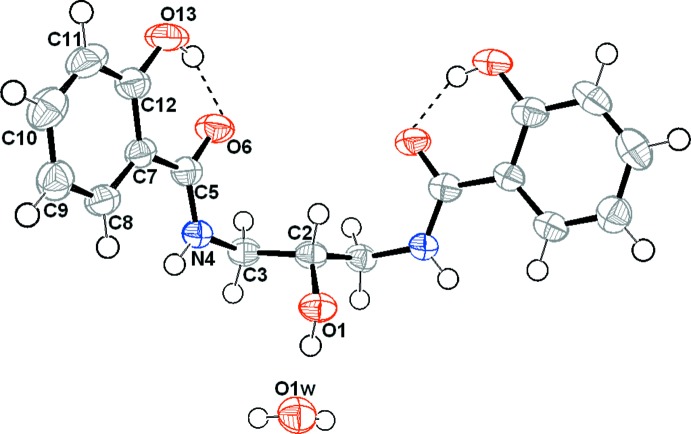
The molecular structure of the title compound with displacement drawn at the 50% probability level. Only the contents of the asymmetric unit are numbered.

**Fig. 2.**
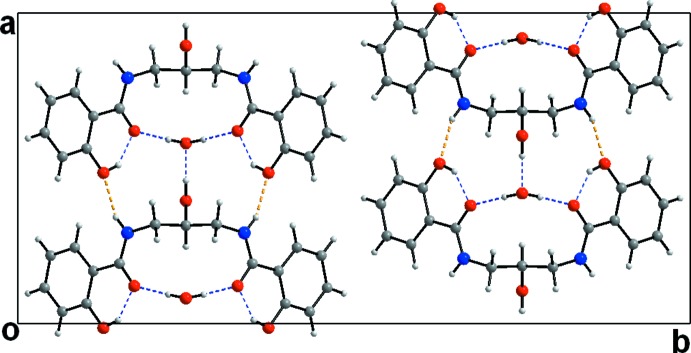
Alternating layers in zigzag parallel to (100) plane of (I) viewed via c axis showing hydrogen bond as dashed line [O—H···O and N—H···O interactions].

## supplementary crystallographic information

### 1. Comment

The chelating agents containing in their molecular structures 2-hydroxy-1,3-diaminopropane appear in a variey of ligands such as salicylamides (Kumar & Debashis, 2006) and Schiff bases (Azam *et al.* 2012) or as mixture of both previous functions in the same molecular structures (Kui *et al.* 2009). This kind of compounds are very attractive and interesting, especially for their coordinating properties with transition metal ions as those involved in metalloenzymes where, for example, the copper complexes are participating in the process of copper transport in humans (Sarkar, 1999). So, the title compound and its analogs are currently used in the synthesis of mono- and polynuclear manganese complexes to investigate their magnetic properties (Louhibi *et al.*, 2007). However, the resulting complexes are, in this case, often bi- or polynuclear owing to their corresponding polydentate nature (NNOOO).

Herein we report the synthesis and crystal structure of *N*,*N*-Bis-Salicylamide(2-hydroxy-1,3-diaminopropane), (I). The molecule structure of (I), and the atomic numbering used, is illustrated in Fig. 1. The asymmetric unit of (I) consists of one-half of the molecule, with the other half generated by a crystallographic mirror plane.

The crystal packing can be described by alterning layers in zigzag parallel to (100) planes (Fig. 2) and the water molecule is sandwished betwen these layers. It features O—H···O and N—H···O hydrogen bonds (Fig. 2, Table 1). A O—H···O intramolecular interaction is also observed. These interactions link the molecules within the layers and also link the layers together and reinforcing the cohesion of the structure.

### 2. Experimental

90 mg (1.0 mmol) of 2-hydroxy-1,3-diaminopropane was dissolved in 10 ml of absolute ethanol and placed in three necked flask of 50 ml. 396 mg (2.0 mmol) of phenylsalicylate were also dissolved in the same solvent (10 ml absolute ethanol). This solution was added in one portion to the previous solution. The resulting mixture, under nitrogen atmosphere and stirring, was heated to reflux for three hours, after which the reaction mixture was filtered as hot solution. Yellow prisms were formed after some days by slow evaporation (yield 70%).

### 3. Refinement

H1W and H1 protons were located in a difference Fourier map and refined isotropically with *U*_iso_(H) = 1.5Ueq(O). The remaining H atoms were localized on Fourier maps but introduced in calculated positions and treated as riding on their parent atom (C,*O* and N) with C—H = 0.99 Å (Methylene)or 0.95 Å (aromatic) or 1.00 Å (methine), O—H = 0.84 Å and N—H = 0.88 Å; with *U*_iso_(H) = 1.2*U*_eq_ and *U*_iso_(H) = 1.5*U*_eq_(hydroxy).

### Crystal data

**Table:**

C_17_H_18_N_2_O_5_·H_2_O	*F*(000) = 736
*M**_r_* = 348.35	*D*_x_ = 1.413 Mg m^−^^3^
Orthorhombic, *P**n**m**a*	Mo *K*α radiation, λ = 0.71073 Å
Hall symbol: -P 2ac 2n	Cell parameters from 951 reflections
*a* = 12.8969 (10) Å	θ = 3.2–21.6°
*b* = 28.001 (2) Å	µ = 0.11 mm^−^^1^
*c* = 4.5330 (4) Å	*T* = 150 K
*V* = 1637.0 (2) Å^3^	Prism, yellow
*Z* = 4	0.38 × 0.12 × 0.04 mm

### Data collection

**Table:**

Bruker APEXII diffractometer	1195 reflections with *I* > 2σ(*I*)
Graphite monochromator	*R*_int_ = 0.062
CCD rotation images, thin slices scans	θ_max_ = 27.4°, θ_min_ = 3.2°
Absorption correction: multi-scan (*SADABS*; Bruker, 2011)	*h* = −16→15
*T*_min_ = 0.877, *T*_max_ = 0.996	*k* = −33→36
7880 measured reflections	*l* = −3→5
1904 independent reflections	

### Refinement

**Table:**

Refinement on *F*^2^	Primary atom site location: structure-invariant direct methods
Least-squares matrix: full	Secondary atom site location: difference Fourier map
*R*[*F*^2^ > 2σ(*F*^2^)] = 0.048	Hydrogen site location: inferred from neighbouring sites
*wR*(*F*^2^) = 0.123	H atoms treated by a mixture of independent and constrained refinement
*S* = 1.04	*w* = 1/[σ^2^(*F*_o_^2^) + (0.0542*P*)^2^] where *P* = (*F*_o_^2^ + 2*F*_c_^2^)/3
1904 reflections	(Δ/σ)_max_ < 0.001
124 parameters	Δρ_max_ = 0.21 e Å^−^^3^
0 restraints	Δρ_min_ = −0.23 e Å^−^^3^

### Special details

**Table:**

Geometry. Bond distances, angles *etc*. have been calculated using the rounded fractional coordinates. All su's are estimated from the variances of the (full) variance-covariance matrix. The cell e.s.d.'s are taken into account in the estimation of distances, angles and torsion angles
Refinement. Refinement of *F*^2^ against ALL reflections. The weighted *R*-factor *wR* and goodness of fit *S* are based on *F*^2^, conventional *R*-factors *R* are based on *F*, with *F* set to zero for negative *F*^2^. The threshold expression of *F*^2^ > σ(*F*^2^) is used only for calculating *R*-factors(gt) *etc*. and is not relevant to the choice of reflections for refinement. *R*-factors based on *F*^2^ are statistically about twice as large as those based on *F*, and *R*-factors based on ALL data will be even larger.

### Fractional atomic coordinates and isotropic or equivalent isotropic displacement parameters (Å^2^)

**Table:**

	*x*	*y*	*z*	*U*_iso_*/*U*_eq_	
O1	0.39517 (14)	0.25000	0.2499 (4)	0.0247 (6)	
O6	0.12018 (10)	0.17317 (5)	−0.0499 (3)	0.0305 (5)	
O13	−0.01478 (10)	0.12722 (5)	0.2259 (4)	0.0365 (5)	
N4	0.29166 (11)	0.16318 (5)	0.0274 (3)	0.0222 (5)	
C2	0.3155 (2)	0.25000	0.0315 (6)	0.0220 (8)	
C3	0.31907 (14)	0.20435 (6)	−0.1551 (4)	0.0231 (6)	
C5	0.19276 (14)	0.15119 (6)	0.0789 (4)	0.0221 (6)	
C7	0.16854 (13)	0.11226 (6)	0.2905 (4)	0.0211 (6)	
C8	0.24447 (14)	0.08491 (6)	0.4318 (4)	0.0246 (6)	
C9	0.21836 (16)	0.04885 (7)	0.6277 (5)	0.0299 (7)	
C10	0.11462 (16)	0.03987 (7)	0.6864 (5)	0.0324 (7)	
C11	0.03789 (16)	0.06622 (7)	0.5520 (5)	0.0324 (7)	
C12	0.06373 (14)	0.10227 (7)	0.3538 (5)	0.0266 (6)	
O1W	0.08072 (15)	0.25000	0.5613 (5)	0.0345 (7)	
H1	0.460 (2)	0.25000	0.145 (7)	0.0370*	
H2	0.24766	0.25000	0.13838	0.0264*	
H3A	0.26982	0.20707	−0.32177	0.0278*	
H3B	0.38959	0.20000	−0.23708	0.0278*	
H4	0.34148	0.14581	0.10545	0.0266*	
H8	0.31554	0.09118	0.39263	0.0295*	
H9	0.27095	0.03049	0.72084	0.0359*	
H10	0.09631	0.01526	0.82091	0.0389*	
H11	−0.03286	0.05978	0.59456	0.0389*	
H13	0.01003	0.14928	0.12199	0.0547*	
H1W	0.0926 (17)	0.2238 (8)	0.677 (6)	0.0517*	

### Atomic displacement parameters (Å^2^)

**Table:**

	*U*^11^	*U*^22^	*U*^33^	*U*^12^	*U*^13^	*U*^23^
O1	0.0203 (10)	0.0348 (11)	0.0189 (11)	0.0000	0.0017 (9)	0.0000
O6	0.0194 (7)	0.0361 (8)	0.0359 (9)	0.0012 (6)	−0.0039 (7)	0.0057 (7)
O13	0.0166 (7)	0.0423 (9)	0.0506 (11)	−0.0004 (6)	0.0023 (7)	0.0076 (8)
N4	0.0185 (8)	0.0223 (8)	0.0258 (10)	0.0002 (6)	0.0017 (7)	0.0005 (7)
C2	0.0183 (13)	0.0282 (14)	0.0196 (15)	0.0000	0.0013 (12)	0.0000
C3	0.0206 (10)	0.0277 (10)	0.0211 (10)	−0.0012 (8)	0.0026 (9)	0.0016 (9)
C5	0.0208 (9)	0.0252 (10)	0.0202 (10)	−0.0002 (8)	0.0012 (9)	−0.0063 (9)
C7	0.0196 (9)	0.0225 (9)	0.0211 (11)	−0.0024 (7)	0.0020 (9)	−0.0043 (8)
C8	0.0205 (10)	0.0272 (10)	0.0262 (11)	−0.0032 (7)	0.0014 (9)	−0.0036 (9)
C9	0.0323 (11)	0.0277 (11)	0.0298 (12)	−0.0008 (9)	−0.0029 (10)	−0.0020 (9)
C10	0.0375 (12)	0.0319 (12)	0.0279 (13)	−0.0092 (9)	0.0060 (10)	0.0005 (10)
C11	0.0263 (11)	0.0357 (12)	0.0353 (13)	−0.0105 (9)	0.0079 (10)	−0.0018 (10)
C12	0.0218 (10)	0.0290 (11)	0.0291 (12)	−0.0010 (8)	0.0009 (10)	−0.0072 (9)
O1W	0.0306 (11)	0.0388 (12)	0.0340 (14)	0.0000	−0.0103 (10)	0.0000

### Geometric parameters (Å, º)

**Table:**

O1—C2	1.427 (3)	C7—C8	1.399 (2)
O6—C5	1.263 (2)	C7—C12	1.410 (2)
O13—C12	1.360 (2)	C8—C9	1.386 (3)
O1—H1	0.96 (3)	C9—C10	1.387 (3)
O13—H13	0.8400	C10—C11	1.377 (3)
O1W—H1W^i^	0.92 (2)	C11—C12	1.392 (3)
O1W—H1W	0.92 (2)	C2—H2	1.0000
N4—C5	1.340 (2)	C3—H3B	0.9900
N4—C3	1.462 (2)	C3—H3A	0.9900
N4—H4	0.8800	C8—H8	0.9500
C2—C3	1.534 (2)	C9—H9	0.9500
C2—C3^i^	1.534 (2)	C10—H10	0.9500
C5—C7	1.485 (2)	C11—H11	0.9500
			
C2—O1—H1	106.4 (18)	C7—C12—C11	120.32 (18)
C12—O13—H13	109.00	O13—C12—C7	121.68 (18)
H1W—O1W—H1W^i^	107 (2)	O13—C12—C11	118.00 (17)
C3—N4—C5	121.76 (14)	O1—C2—H2	107.00
C3—N4—H4	119.00	C3^i^—C2—H2	107.00
C5—N4—H4	119.00	C3—C2—H2	107.00
O1—C2—C3^i^	111.17 (13)	N4—C3—H3B	110.00
O1—C2—C3	111.17 (13)	C2—C3—H3A	110.00
C3—C2—C3^i^	112.93 (19)	N4—C3—H3A	110.00
N4—C3—C2	109.75 (15)	H3A—C3—H3B	108.00
O6—C5—N4	120.19 (16)	C2—C3—H3B	110.00
N4—C5—C7	119.79 (15)	C7—C8—H8	119.00
O6—C5—C7	120.01 (16)	C9—C8—H8	119.00
C5—C7—C12	118.60 (16)	C10—C9—H9	120.00
C8—C7—C12	118.00 (16)	C8—C9—H9	120.00
C5—C7—C8	123.40 (15)	C9—C10—H10	120.00
C7—C8—C9	121.48 (17)	C11—C10—H10	120.00
C8—C9—C10	119.29 (19)	C10—C11—H11	120.00
C9—C10—C11	120.75 (19)	C12—C11—H11	120.00
C10—C11—C12	120.15 (19)		
			
C3—N4—C5—O6	5.8 (3)	C12—C7—C8—C9	0.3 (3)
C3—N4—C5—C7	−173.61 (15)	C5—C7—C12—O13	0.2 (3)
C5—N4—C3—C2	83.3 (2)	C5—C7—C12—C11	−179.68 (18)
O1—C2—C3—N4	67.2 (2)	C8—C7—C12—O13	179.97 (19)
C3^i^—C2—C3—N4	−167.07 (16)	C8—C7—C12—C11	0.1 (3)
O6—C5—C7—C8	177.11 (17)	C7—C8—C9—C10	−0.4 (3)
O6—C5—C7—C12	−3.1 (3)	C8—C9—C10—C11	0.2 (3)
N4—C5—C7—C8	−3.5 (3)	C9—C10—C11—C12	0.2 (3)
N4—C5—C7—C12	176.29 (17)	C10—C11—C12—O13	179.8 (2)
C5—C7—C8—C9	−179.93 (17)	C10—C11—C12—C7	−0.4 (3)

Symmetry code: (i) *x*, −*y*+1/2, *z*.

### Hydrogen-bond geometry (Å, º)

**Table:**

*D*—H···*A*	*D*—H	H···*A*	*D*···*A*	*D*—H···*A*
O1—H1···O1*W*^ii^	0.96 (3)	1.82 (3)	2.778 (3)	179 (3)
O1*W*—H1*W*···O6^iii^	0.92 (2)	1.92 (2)	2.827 (2)	174 (2)
O13—H13···O6	0.84	1.75	2.500 (2)	147
N4—H4···O13^iv^	0.88	2.07	2.915 (2)	160

Symmetry codes: (ii) *x*+1/2, −*y*+1/2, −*z*+1/2; (iii) *x*, *y*, *z*+1; (iv) *x*+1/2, *y*, −*z*+1/2.
